# Effect of Product Involvement on Panels’ Vocabulary Generation, Attribute Identification, and Sample Configurations in Beer

**DOI:** 10.3390/foods8100488

**Published:** 2019-10-12

**Authors:** Line Elgaard, Line A. Mielby, Hildegarde Heymann, Derek V. Byrne

**Affiliations:** 1 Department of Food Science, Faculty of Science and Technology, Aarhus University, DK-5792 Aarslev, Denmark; lineh.mielby@food.au.dk (L.A.M.); derekv.byrne@food.au.dk (D.V.B.); 2 Department of Food Science and Technology and Viticulture & Enology, University of California, Davis, CA 95616-5270, USA; hheymann@ucdavis.edu

**Keywords:** sensory descriptive analysis, vocabulary generation, attribute learning, product involvement, beer

## Abstract

The aim of this study was to compare the performance of two semi-trained panels with different degrees of self-reported beer involvement in terms of beer consumption pattern. The two panels were beer non-drinkers (indicating willingness to taste beer) and craft-style beer drinkers. Eleven modified beer samples were evaluated during three separate tasks by both panels. The tasks were (1) a vocabulary generation on a sample level, (2) an attribute identification task with a list of attributes to choose from, and (3) a descriptive analysis. The performance of the two panels was evaluated and compared using three parameters, as follows: Descriptive similarity, attribute knowledge similarity, and perceptual similarity. The results showed that the craft-style beer drinkers generated the most precise vocabulary and correctly identified more attributes, compared to the beer non-drinkers. Furthermore, the sample sensory spaces generated by the two panels were different before the training period, but were perceptually similar post training. To conclude, the beer consumption pattern influenced all aspects of panel performance before training, with the craft-style panel performing better than the non-drinkers panel. However, the panels’ performance became more similar after a short period of training sessions.

## 1. Introduction

Many different methods and approaches exist within the field of sensory science. However, sensory descriptive analysis (DA) remains an essential and crucial method in the toolbox of a sensory scientist, when dealing with e.g., product development or quality control [1,2,3,4]. The drawbacks of DA are that it is a slow and cost intensive method, which has resulted in the recent focus on so-called rapid methods [5,6,7]. The shift in focus has caused a decrease in research on the methodology of DA methods during the last decades [8], even though validation and improvement of the DA method is still necessary today [1,4].

The largest advantage of rapid methods is that they can be executed by both trained and untrained panelists [6], while DA requires a training period for the panelists [1,2,4]. An extensive amount of research has been done comparing the performance of panelists with different degrees of sensory training in both DA and rapid methods [9,10,11,12,13,14,15]. Generally, the results of these studies show that the generated sensory configurations are somewhat similar, but that sensory training increases the panels’ ability to discriminate verbally between the products. The same is the case for vocabulary generation where studies have shown that the panelist’s level of sensory training influences the ability to generate vocabularies [11,12,16,17,18]. However, regardless of the type of sensory method, rapid or DA, a recruitment process is needed to select panelists before a potential training period can be carried out. The question is then, whom to recruit? Studies have shown that the discriminability of new panelists is affected by factors like age, gender, personality traits, and lifestyle [19,20,21,22,23,24,25,26,27]. It is also suggested that food involvement could influence the discriminability of panelists [28]. Food involvement can either be measured by the food involvement scale (FIS, [28]) or by asking the panelists about their self-reported food involvement. Both Gains and Thomson (1990) and Bell and Marshall (2003) suggest that panelists who have a higher food involvement could potentially be better at discriminating between food products, based on a higher attention during interaction with these food products [28,29]. Bell and Marshall (2003) tested this by comparing the discriminability of participants with different FIS scores and found that there was indeed an association between FIS score and discriminability [28]. Giacalone et al. (2016) also compared the discriminability of two panelists groups with different self-reported food involvement (novices and enthusiasts) evaluating beer products [13]. They found that both groups perceived the beers similarly and that they yielded similar configurations in the sensory space. This indicated that the self-reported product involvement did not influence the perceptual ability in their study. However, they argue that this could be because of the very diverse sensory span of their samples. Comparing the groups vocabulary generation showed that the novice group used a more holistic, abstract, and emotional vocabulary. Thus, the product involvement was more important for the verbalization of sensory perception, as opposed to perceptual ability. Further, Vidal et al. (2015) studied the influence of wine involvement on the ability to generate terms and describe wine astringency [30]. They found no significant difference between groups with varying wine involvement for the descriptions of wine astringency, but there was a significant difference in the number of astringency terms generated by the two groups, with high wine involvement resulting in a higher number of terms. Lastly, Byrnes et al. (2015) compared the generation of perceptual maps and the vocabulary of chemesthetic stimuli for two groups of untrained panelists with a low and high FIS score, respectively [31]. The perceptual maps generated by both groups were significantly similar and there was no significant difference in the number of attributes generated. In summary, the results of the above studies are inconclusive regarding the influence of food involvement (either measured by FIS score or self-reported involvement) on the discriminability of products, which is why this effect (both perceptual and descriptive discriminability) should be studied further.

The aim of this current paper is therefore to compare the performance of two semi-trained panels with different degrees of self-reported beer involvement in terms of consumption pattern (beer non-drinkers versus beer craft-style drinkers), before and after a training period. The panels’ performance level will be evaluated on the three following criteria: (1) Descriptive similarity, the ability to describe the sensory experiences verbally, i.e., vocabulary generation, (2) attribute knowledge similarity, the ability to correctly identify beer specific attributes, and (3) perceptual similarity, the ability to discriminate between the beers in the sensory space. These evaluation criteria were inspired by Giacalone et al. (2016) [13].

## 2. Materials and Methods

### 2.1. Experimental Overview

**Figure 1:**
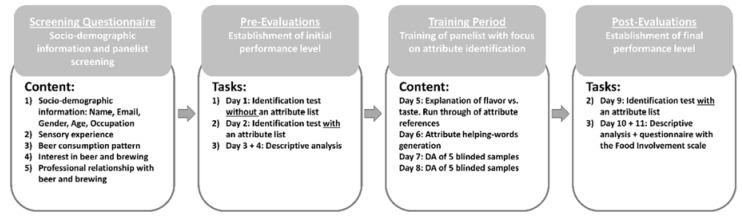
Overview of the experimental parts: Screening questionnaire, pre-evaluations, training period, and post-evaluations. The content/tasks during each step is described in the boxes.

An experimental overview of the study is shown in Figure 1 and each activity is described in detail throughout the materials and methods section. The study started out with a screening questionnaire, including sociodemographic information and information about beer consumption patterns, for the selection of panelists. When the two groups of panelists were identified, they performed three pre-evaluation tasks to establish the panelists’ initial state of performance. The three tasks were (1) an attribute identification test, without a list of attributes to choose from, i.e., vocabulary generation, (2) an attribute identification task with a list of attributes to choose from, i.e., attribute knowledge and identification, and (3) a descriptive analysis, i.e., scaling of attribute intensity and sensory configurations. After the two panels had conducted the pre-evaluation tests, they were trained in daily one-hour sessions on four consecutive days. Lastly, tasks number one and two were conducted again (post-evaluations) in an identical manner to the pre-evaluations. Comparing the panels’ evaluations before (pre) and after (post) the training period, gave insight into the performance difference based on beer consumption habit and how the training session affected the two panels’ performance.

### 2.2. Screening Questionnaire

A screening questionnaire (Figure 1) was used to identify panelists with two different beer consumption habits, i.e., beer non-drinkers and craft-style drinkers. The beer non-drinkers panel consisted of panelists who indicated that they did not drink beer on a regular basis, but that they were willing to try it. The requirement for becoming part of the craft-style panel was that the panelists indicated that they drank at least three of the four presented craft-style beers. An overview of the questions included in the screening questionnaire and the answer options is provided in the supplemental materials Appendix A. Eliminated participants included people who were not willing to taste beer, people who worked professionally with beer, and people who were either exclusively light-style drinkers or drank both light- and craft-style beers. The participants had to be of the legal drinking age (21 years in US) to participate. The beers chosen as light-style and craft-style beers in the questionnaire were representatives of the local market.

### 2.3. Samples

A total of 11 model beer samples were evaluated in duplicates. The beer samples were all modifications with the Carlsberg pilsner (Carlsberg Brewery, Fredericia, Denmark) as base and spiked with various flavor capsules from AroxaTM and Isohop® (Iso-α-acids: 30% *w*/*w*, Barth-Haas Group). An overview of the different samples and the modifications are presented in Table 1. The modifications were chosen based on knowledge from a previous study (Reference [32] in review). The Carlsberg beer was purchased in 50 L kegs and stored at 0 °C until the day of evaluation, were it was placed in a tempered 5 °C kegerator (Edgestar KC3000). The Carlsberg beer was tapped into Pyrex® bottles and mixed with flavor capsules or Isohop®, and stored at 6 °C until serving. For all tasks, the model beers were prepared one hour prior to the first evaluation (not all panelists evaluated the samples at the same time, but were parted into different evaluation sessions) and a new batch was made halfway through the number of evaluation sessions, to account for the loss of sensory quality, such as carbonation. During each evaluation session, all samples were poured simultaneously to obtain a similar loss of carbonation and increase in temperature between samples. The serving temperature therefore ranged between 13 °C to 22 °C from the first evaluated sample to the last. The influence of the temperature range was minimized by serving the samples in a randomized order and, furthermore, the same procedure was applied for both panels, why the influence of serving temperature was similar between panels. The samples (30 mL) were served under red light and in black wine glasses (Riedel Blind Tasting Glasses, #8446/15, 13 oz) with a clear plastic lid (Dart/Solo® Ultra Clear Container Lids, #PL4N). The use of red light and black wine glasses were chosen to mask the lack of visual difference between the samples, so that the nature of the samples (model system) was not clear to the panelists. The samples were served 5–6 at the same time and panelists were instructed not to go back and forth between the samples. The serving order was randomized for all tasks using a Latin square design. Water (Arrowhead spring water) and crackers (Nabisco Premium Saltine Crackers, unsalted tops) were available as palate cleaners. Due to the presence of alcohol in the samples, the panelists were required to expectorate to avoid intoxication.

### 2.4. Panels

The study was conducted at the Robert Mondavi Institute, University of California, Davis, USA and the participants were individuals from the surrounding area. The study was approved as exempt by the University of California, Davis IRB board (study #1321096-1). The two panels, craft-style drinkers and beer non-drinkers, were treated as one big panel of 15 panelists during both training and evaluation sessions. However, for the statistical analysis, the panels were analyzed individually. The craft-style panel had seven participants (four females, age average: 30.7 years) and the non-drinkers panel had eight participants (five females, age average: 29.5 years).

### 2.5. Pre-Evaluations

The pre-evaluations (Figure 1—Pre-Evaluations, task 1–3, day 1–4) were conducted to establish the panelists’ initial performance level for comparison between the two panels, and to create a baseline with which to compare the post-evaluations. The pre-evaluations consisted of three individual tasks on four consecutive days.

#### 2.5.1. Identification Test without an Attribute List (Task 1)

The first task on the first day was an identification test without a list of attributes. For each sample, the panelists were asked to smell/taste the sample and indicate the single most intense attribute that they experienced. Since they did not have a list of attributes to choose from, they were free to write whatever came to their mind. The aim of this task was to give insight into the panels’ ability to generate a sensory vocabulary. The panelists were served all 11 samples in duplicates (22 in total) with a 30 sec fixed break between samples. Furthermore, there was a 2 min break after the 6th and 17th sample and a 3 min break after the 11th sample.

#### 2.5.2. Identification Test with an Attribute List (Task 2)

The second task on the second day, was similar to task (1). However, this time the panelists had a predefined list of attributes to choose from, with the attribute options of hoppy, malty, sulfury, fruity, and bitter. They were again instructed to smell/taste the sample and choose the single most intense attribute for each sample. The aim of this task was to establish the panels’ attribute knowledge and ability to identify different beer related attributes. The order of the attribute list was randomized between panelists, but fixed for each panelist. The panelists were served all 11 samples in duplicates (22 in total) with a 30 sec fixed break between samples. Furthermore, there was a 2 min break after the 6th and 17th sample and a 3 min break after the 11th sample.

#### 2.5.3. Descriptive Analysis (Task 3)

The third task on the third and fourth day, was a descriptive analysis (DA) test (without any prior training). The aim of this task was to compare the panels’ ability to scale the different beer related attributes and generate sensory configurations, i.e., perceptual similarity. The 11 beer samples were again tested in duplicates and because of the large number of samples, the DA was separated into two consecutive days. Half of the samples, including replicates, were evaluated on day 3 and the other half of the samples were evaluated on day 4. The samples were evaluated on a 15 cm line scale labeled with “a little” to “a lot” at the endpoints using FIZZ (version 2.61.0, Biosystèms, Couternon, France). The attributes included the aroma and flavor of hoppy, sulfury, malty, and fruity, together with the basic taste of bitter.

### 2.6. Training Period

After the two panels had performed the three pre-evaluation tasks (attribute identification with and without a list and DA), they were trained during four one-hour sessions on four consecutive days (Figure 1—Training Period, day 5–8). The total number of panelists was too great to train all panelists simultaneously, therefore there was two separate training sessions conducted during each day. The panelists could choose randomly between the two training sessions during each day and the panelists were therefore mixed between the two panels during each training session. This was done to ensured that the performance of the two panels was not affected by “time of day” or “training session”. The focus during the first session was on teaching the panelists the difference between the attribute flavors, and, furthermore, to teach them the difference between taste and flavor. This was done since the samples were expectorated and the panelists therefore needed to focus more on the development of flavor in their mouth/nose. They were trained to blow air through their nose after expectoration, to force some of the flavor compounds through the retro nasal pathway and into the nose. The teaching of attribute flavors was done by serving references (high intensity version of model samples with attribute labels) to the panelists. The second session consisted of panelists generating their own attribute helping words followed by a plenary discussion to further facilitate the attribute understanding and panel alignment. The third and fourth training sessions included a training descriptive analysis of five blinded samples, together with a run-through of the individual panelists’ results. This exercise gave rise to further plenum discussion about attribute understanding and scale use.

### 2.7. Post-Evaluations

The two post-evaluation tasks (Figure 1—Post-Evaluations, day 9–11) were conducted after the training sessions to establish the panels’ performance level on each task after a training period. The post-evaluations did not include task (1), the identification test without an attribute list, as the panelists were already familiar with the list of attributes. However, it did include task (2) the identification test with an attribute list and task (3) the DA. Both the procedure for task (2) and task (3) were identical to the ones performed during the pre-evaluations. The study was concluded by a short questionnaire including the Food Involvement Scale (FIS, [28], Appendix B), however, this was not connected to the any of the tasks itself, but was placed in the end of the post-evaluations, to avoid informing the panelists about the aim of the study.

### 2.8. Statistical Analysis

Statistical analysis was performed in R (Version 3.5.3, R Core Team, Vienna, Austria) using RStudio (Version 3.4.2, RStudio Inc., Boston, MA, USA).

#### 2.8.1. Comparison of the Panels’ Vocabulary Generation—Identification Test without a List

First, the words generated by the panels were separated into semantic attribute groups based on indications of similar sensory sensations (e.g., “pine”, “pine tree”, “hoppy”). This was done through a discussion among researchers. This resulted in 16 semantic attribute groups with different sensory themes. Second, a correspondence analysis (CA) was performed to visually analyze the correlation between frequencies for the two categorical variables *product* (11 levels, one for each product) and *attribute group* (16 levels, one for each sematic attribute group described above), i.e., the panels’ descriptive similarity. The data was transformed into a contingency table and the CA was performed with chi-squared distances applied using the epCA function from the ExPosition package [33]. The biplot was created with the fviz_ca_biplot function from the factoextra package [34].

#### 2.8.2. Comparison of the Panels’ Identifications of Attributes and Attribute Understanding—Identification Test with a List

The results from the identification test with a list (i.e., attribute knowledge similarity) was obtained as count numbers. These count numbers for each sample and answer category (attribute) were then averaged over the panelists and calculated into percentages of panelists choosing the specific answer category. The percentages were then plotted with the mosaic function from the vcd package [35]. The panels’ performance enhancement was compared by investigating the frequency of correct/incorrect answers between panels both before (pre) and after (post) training, respectively. Furthermore, the performance within each panel was compared before (pre) and after (post) training, to evaluate the performance enhancement within panels. The significance of the results was tested with two different tests, namely Pearson’s chi-squared with the Yates correction to account for low cell count numbers and the Wilcoxon signed-rank test. The chi-squared test was used for the significance testing between panels, while the Wilcoxon test was used for the significance tests within panels (i.e., comparing results before and after training). The Wilcoxon test is a nonparametric test, which is well suited to test significance of repeated measurements for the same sample. For both tests, the count numbers were converted into frequencies of correct/incorrect answers for each panelist. The count numbers were averaged over the panelists for the chi-squared test, and over samples for the Wilcoxon test. This results in the frequencies being tested were on a sample by sample basis (not for the control sample, as it does not have a “correct” answer) for the chi-squared test and on a panelist basis for the Wilcoxon test. The chi-square tests were conducted with the chisq.test function from the stats package [36] and the Wilcoxon signed-rank test was performed by the wilcoxsign_test function from the coin package [37] with zero.method equal to Wilcoxon.

#### 2.8.3. Comparison of the Panels’ Positioning of the Samples in the Sensory Space—Sensory Profiling Data

A Generalized Procrustes analysis (GPA, [38]) was performed to visually compare the panels’ positioning of the beer samples in the sensory space, i.e., perceptual similarity. The data was averaged over both replicate and panelist. The GPA was performed by the GPA function from the FactoMineR package [39]. The tolerance level was set to 10–10, the maximum number of iterations was set to 200, and the data was scaled to unit variance. A permutation test [40,41] was performed with the GPA.test function from the RVAideMemoire package [42], to investigate the significance based on the total variance explained from the GPA. The number of permutations was 500. Furthermore, RV coefficients were calculated from the GPA to investigate the panels’ similarities/differences based on a numerical value.

#### 2.8.4. Comparison of the Panels’ Individual and Average FIS Scores

The total FIS score for each panelist and the panel average scores were calculated according to [28] by first reversing the scores of item number 1, 2, 4, 8, 9, and 11. Then, the score for each item for each panelist was summed, resulting in a total FIS score, with the possible range of 12 to 84, and the individual scores were plotted as a bar graph. A student’s t-test was performed to test the significance difference in average score by the two panels.

## 3. Results

### 3.1. Comparison of the Panels’ Vocabulary Generation—Identification Test Without a List

**Figure 2:**
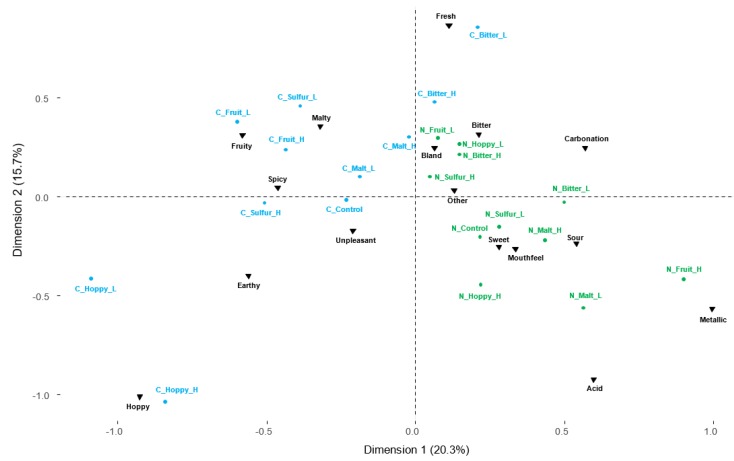
Correspondence analysis from identification test without an attribute list. Answers are grouped into themes (black triangles). The craft-style group answers are colored in blue. Non-drinkers are colored in green.

The correspondence analysis (Figure 2) based on the identification test without a list (Figure 1, task 1) shows a clear separation between the two panels. The craft-style panel has a more precise and specific vocabulary, with fruity, hoppy, and malty samples placed closer to the fruity, hoppy, and malty descriptors, respectively, compared to the non-drinkers. On the contrary, it seems that the describing words generated by the non-drinkers are more related to basic tastes (i.e., bitter, sweet, and sour) and more abstract terms like bland, mouthfeel (i.e., full, thick) and other (i.e., dark, aromatic, unusual). This indicated that the panels had different sensory descriptive abilities, with the craft-style panel being more precise in their vocabulary generation and thereby better at describing and identifying the flavors in the beer samples.

### 3.2. Comparison of the Panels’ Identifications of Attributes and Attribute Understanding—Identification Test with a List

**Figure 3:**
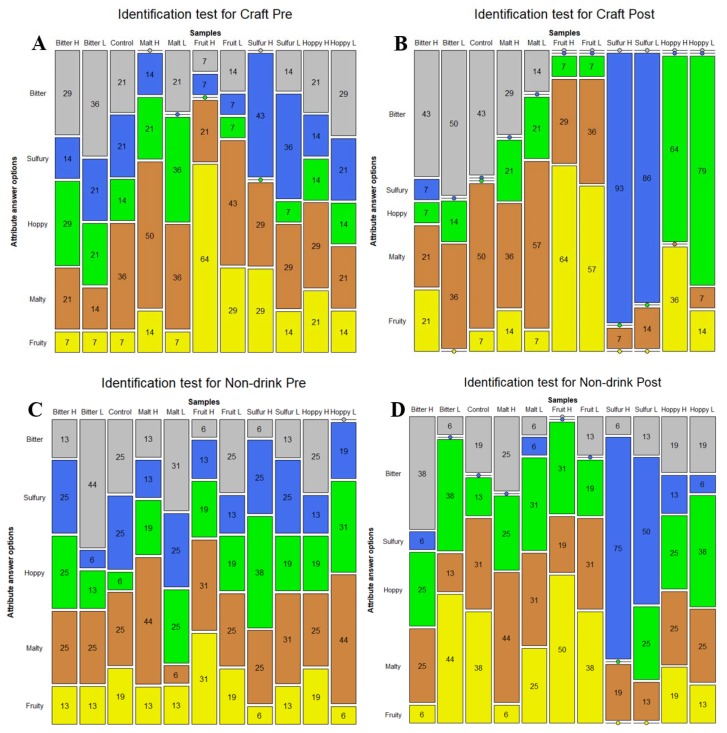
Mosaic plot for percentages of replies during the identification test with an attribute list. The color codes are as follows: grey = bitter, blue = sulfury, green = hoppy, brown = malty, and yellow = fruity.

The results from the mosaic plots (Figure 3) show the percentages of answers for the different attribute options for each sample. The plot (Figure 3A) for the identifications for the craft-style panel before training shows that the easiest sample to detect was the high fruity sample, with 64% correct answers, followed by the high malty sample, with 50% correct answers. The hoppy samples received the lowest percentage of correct answers, with only 14% for both samples. In general, the panelists had trouble identifying the correct attributes when the flavor concentrations were low. The high bitter sample was often confused with the hoppy sample, while the hoppy samples were thought to be malty or bitter. The low malty sample was confused with a hoppy flavor, while the low fruity sample was confused with the malty flavor.

The percentages for the non-drinkers panel before training (Figure 3C) shows that the samples with the highest percentage of correct identifications are malt high (44%) and bitter low (44%), while on the contrary, the samples with the lowest identification percentages were malt low (6%) and bitter high (13%). For the craft-style panel, there was an overall trend that low intensity flavors were less often identified. However, this was not observed for the non-drinkers. However, the non-drinkers more often confused the attributes malty, hoppy, and bitter with one-another. Compared to the craft-style drinkers, the non-drinkers had a lower percentage of correct identified samples for all samples, except for bitter low, hoppy low, and hoppy high. This indicates that the craft-style drinkers were better at identifying the correct attributes.

The mosaic plot for the two panels after training (Figure 3B,D) shows that both panels increased their percentage of correct identifications for all samples except for bitter low and malty high for the non-drinkers, and malty high and fruity high for the craft-style drinkers. It is noteworthy that for both panels, the samples without an increase were also the samples for which both panels had the highest percentage of identification before the training sessions. Furthermore, the craft-style panel had the highest percentage of correct identifications for all samples except malty high, again indicating that the craft-style panel was better at identifying the attributes. The craft-style panel increased their percentages of correct scores markedly, especially for the sulfury and hoppy samples, while some confusion still existed for the bitter and malty samples. The non-drinkers panel increased their percentages of correct scores, especially for the sulfury samples, while some confusion still existed for the hoppy samples and generally for the low flavor intensity samples.

The results from the chi-squared tests between panels (Table 2) show that only one of the chi-squared tests was significant, while one test was close to significance (*p* = 0.058). The significant differences were for the frequencies of correct answers after the training sessions for bitter low, and borderline for the hoppy low sample. This indicates that, even though differences in attribute identification were found between panels before or after training, these differences were most often not significant. The results from the Wilcoxon test showed that the number of correct frequencies increased significantly between pre-test and post-test for the craft-style panel (Z = −2.2, *p* = 0.028), while the increase was not significant for the non-drinkers panel (Z = −1.7, *p* = 0.09).

### 3.3. Comparison of the Panels’ Positioning of the Samples in the Sensory Space—Sensory Profiling Data

**Figure 4:**
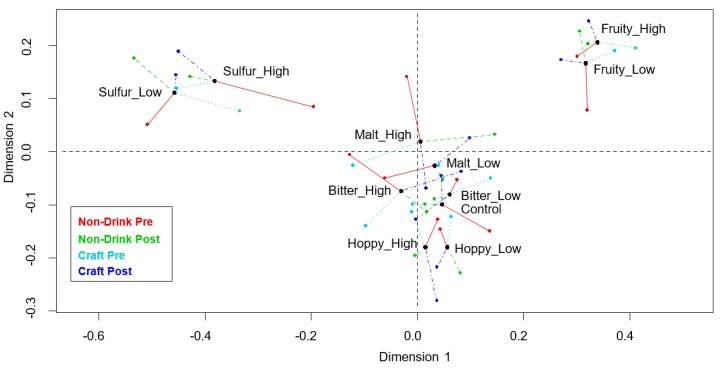
GPA plot for sensory profiling data pre and post training sessions, for both the craft-style panel (pre is light blue and post is dark blue) and non-drinkers panel (pre is red and post is green).

The General Procrustes analysis (GPA) plot (Figure 4) shows that, generally, all samples with the same flavor modifications placed close together (e.g., hoppy low and high), indicating that the samples with similar flavor modifications were also perceived similarly. Furthermore, the plot shows that the sulfury and fruity samples were well discriminated from each other and from the remaining samples, which were found to be more similar. The control sample was placed closest to the low bitterness sample, followed by the low malty and low hoppy samples. A permutation test was calculated and the p-value was significant (*p* < 0.001), indicating that the GPA model was meaningful, i.e., significantly different from what could be obtained randomly. Looking at the individual panel means compared to the consensus mean, shows that there is no system in which the panel was closest to the consensus mean for the different samples. The RV-coefficients, calculated from the GPA, show that the two most similar sample sensory spaces were the craft-style drinkers post training and the non-drinkers post training, with an RV-coefficient of 0.93. For comparison, the RV-coefficient for both panels before training was 0.73. When comparing the sensory space within panels for the non-drinkers and craft-style drinkers before and after training, the RV-coefficients were 0.77 and 0.79, respectively. The least similar sensory configuration (RV = 0.69) was between the non-drinkers before training and the craft-style drinkers after training. Lastly, the comparison between the sensory space generated by the non-drinkers post training and the craft-style panel before training was 0.77.

### 3.4. Comparison of the Panels’ Individual and Average FIS Scores

The panelists’ individual FIS scores (data not presented visually) ranged from 62 to 80, which is on the higher end compared to the possible span of 12 to 84 for FIS scores. The craft-style panelists had the highest FIS scores, with an average score of 72.1, while the non-drinker panelists had the lowest FIS scores, with an average score of 69.8. However, there was no significant difference in average FIS score between the two panels (*p* = 0.376).

## 4. Discussion

### 4.1. Comparison of the Panels’ Descriptive Similarity—Vocabulary Generation

Correspondence analysis showed that the craft-style panel had a more precise vocabulary compared to the non-drinkers, indicating a higher descriptive ability. These results are in agreement with the results of Giacalone et al. (2016), who also found that product involvement increased the panels’ ability to describe the samples using a more precise vocabulary [13]. On the contrary, both Vidal et al. (2015) and Byrnes et al. (2015) did not find a difference in verbal discrimination [30,31]. The differences in results could be a consequence of the differences in products and methodology, since all of these studies used different products (i.e., beer, wine, chemesthetic stimuli) and different methods (napping, DA, CATA, sorting). The use of different products can alter the task complexity by having more or less complex products or a more narrow/broad product sensory range. Furthermore, the differences in methodology also add differences to the complexity, as a rapid method related tasks (i.e., CATA, napping sorting) is simpler to perform compared to DA.

### 4.2. Comparison of the Panels’ Attribute Knowledge Similarity—Identification of Attributes

The results from the identification test before training (Figure 3A,C) showed that the craft-style panel had the overall highest percentage of correct identifications. This demonstrates that, without any prior panel training, a higher food involvement is associated with a higher attribute knowledge. Both panels had the higher levels of correct identifications for the high malty and fruity samples, indicating that these attributes were easier for the panels to identify compared to the other attributes. Furthermore, the craft-style panel also had a higher identification rate for the high sulfur sample, while the non-drinkers panel had a higher identification rate for the low bitter sample. However, it is noteworthy that both panels had the highest percentage of correct identifications for the highly malty sample before training, while at the same time, both panels also most often indicated a malty flavor for samples that were not spiked with malty flavor. This indicates that the panels confused malt with other flavors. This gives thoughts to whether the panels actually were able to distinguish malt from the other flavors, or if malt was used as the default answer for attributes that the panelists could not identify. Malt could be used as the default answer because a generally strong flavor in beer was associated with a high concentration of the important ingredients in beer (i.e., malt). This way, malt could receive higher scores when it was indeed malt, because malt was used as a “default” answer and not because malt was identified. This theory is further supported by the fact that none of the panels increased their identification percentages for the highly malty sample after the training session. For the hoppy samples, the overall identification rate before training was low for both panels, but especially for the craft-style panel. Additionally, hoppy was the second most frequent attribute chosen by both panels in cases of wrong identifications, indicating that some confusion also existed for this attribute. On the other hand, fruity and sulfury were the least mentioned attributes for both panels, in cases where wrong attributes were picked. Overall, the results from the attribute identification test correlate well with the results from the configurations of sample sensory space (DA), showing that the samples associated with fruity and sulfur were the ones with best discrimination compared to the other samples, i.e., hoppy, bitter, and malty (GPA plot, Figure 4).

The reason why hoppy and malty flavors are more difficult to identify could be because they are more product specific compared to sulfury and fruity. Hoppy and malty are most often encountered together and in the same context of beer. It is therefore possible that learning and distinguishing these flavors would be more difficult, because one never experiences them separately. Chollet and Valentin (2001) discussed something similar, namely that more common, familiar, and daily encountered flavors may be easier to identify, compared to uncommon and unknown flavors that are not encountered daily [16].

The results after the training sessions showed that the craft-style panel still had the highest percentage of correct identifications for almost all attributes. The results also show that the training period indeed did increase both panels attribute knowledge, as the percentage of correct identified attributes increased after training for both panels. Nevertheless, both panels still had imperfect results and more training was still needed to teach the panelists the difference between the attribute. The craft-style panel increased their percentages of correct scores markedly for sulfur and hoppy, indicating that it is possible to teach the panelists an unfamiliar attribute in a short number of training sessions. Since the non-drinker panel did not obtain as high identification scores compared to the craft-style panel, it must be concluded that training a panel with regards to attribute identification requires less time for a high food involvement panel, compared to training a panel with low food involvement.

In general, there was only one chi-squared test which was significant between the panels, and only the test for the craft style panel was significant within the panels. Only one significant difference was obtained between the panels (i.e., the difference in attribute knowledge among panels), while the largest difference seems to be found within panels. This shows that the training effect was more important than the level of food involvement between panels, as training seemed to influence the number of correct identifications more. However, it can still be argued that the choice of panel is important, as the craft-style panel was the only one with a significantly increased number of correct identifications (when comparing before and after training), indicating that training had the largest effect on this panel.

### 4.3. Comparison of the Panels’ Perceptual Similarity—Sample Positioning in the Sensory Space

Comparing the RV coefficients of the two panels’ sample configurations before and after training showed that their configurations became markedly more similar after the training session (0.73 vs. 0.93, respectively). This indicates that the short training period in this study equalized the perceptual difference between the panels and that the choice of panel therefore became indifferent with regards to perceptual ability.

The results of previous studies showed varying influences of food involvement on perceptual ability. Bell and Marshall (2003) found that food involvement increased the perceptual ability [28], while both Giacalone et al. (2016) and Byrnes et al. (2015) did not find an effect of food involvement [13,31]. This difference in outcomes correlates with the results of the current study, which showed differences in the sample sensory space generated between the two panels before training but not after training. The reason why we see these results could be based on the complexity of the task, since the current results changed with training. Both Giacalone et al. (2016) and Byrnes et al. (2015) used samples with a large sensory span [13,31], while the current study and Bell and Marshall (2003) used samples with a smaller sensory span [28]. A narrower sample sensory span increases the task complexity and requires the panelist to have a more sensitive perceptual discriminability. This issue was also touched upon by Giacalone et al. (2016), who argue that their results may not be transferrable to studies with a smaller sensory span [13]. The results therefore indicate that food involvement is more important if the sample sensory span is narrow, i.e., the task complexity is high.

The results of the current study have implications for scientists working with both fast and slow methods. For fast methods (e.g., napping or sorting), when the aim is to obtain as precise configurations and descriptions as possible, the choice of panelist is dependent on whether or not a training period is applied. If a training period is applied, then the choice of panelist is less important, however, if no training is applied, then the scientist should choose panelists with a higher level of food involvement. The aim of a slow method like DA is to obtain as precise sensory descriptions as possible and therefore a training session will always be applied in these cases. Therefore, when considering what type of panel to choose for a sensory study, one should compare the results after the training period (post training). The results after training showed that the panels’ perceptual ability was similar, but that the high food involvement panel (craft-style panel) had the highest percentage of correctly identified attributes. This indicates that a high food involvement panel is advantageous. Furthermore, the results from the panels’ attribute knowledge, descriptive, and perceptual ability, before training, also indicated that selection of panelists with a high food involvement would be favorable. One could argue that the choice of panel would be evened out by a more extensive amount of training, as the panels would become more similar after training. However, more extensive training means spending more time and, hence, money, which is not beneficial for the researchers. A high food involvement panel is therefore recommended.

### 4.4. Comparison of the Panels’ Individual and Average FIS Scores

The results from the individual panelists’ FIS scores showed that the panelists included in the current study all had high FIS scores (ranged from 62 to 80), while the general FIS scores found by Bell and Marshall (2003) ranged from 14 to 78 [28] (the possible FIS score span is 12–84). This indicates that all panelists who participated in the current study had a high general food involvement. This might be because people who are more interested in foods are more likely to participate in a sensory study, as participation does require some commitment from the panelist. Thus, people with low food involvement are probably not food interested enough to commit to participating in a three-week long study. This resulted in panelists with large FIS values and, thus, a narrow FIS score span, which is why no significant difference was found between the panels’ average FIS score. This is surprising, since the panelists were selected based on their difference in food involvement, i.e., beer drinking habit (non-drinker versus craft-style drinker). One could therefore ask the following question: How can the two panels have similar FIS scores but at the same time have different beer drinking habits?

A probable reason could be that there is a difference in what the FIS score measures and what the panel selection criteria (drinking habits) measures. The FIS score is concerned with the overall food involvement, while the information about beer drinking habits is concerned with the food involvement on a product level (i.e., beer). This creates the opportunity for a panelist to have a high general food involvement, but at the same time, have a low food involvement for beer in particular.

The results of the current study showed a difference between the two panels in vocabulary generation, attribute identification, and generated sensory configurations before training. As this difference in performance was measured before training, it must be a result of the difference in the panels’ level of food involvement. Still, since the panels had no difference in average FIS score, but did have a difference in beer drinking habit, this difference in performance must be based on the differences in drinking habits (product level food involvement). This suggests that food involvement on a product level is more important for a panel’s performance than food involvement on a general level. It is therefore suggested that future studies consider food involvement on a product level as opposed to on a general level, when investigating the influence of food involvement on panel performance.

## 5. Conclusions

The current study found a difference in descriptive ability between two different sensory panels, since the craft-style panel generated a more specific and precise vocabulary, compared to the non-drinker panel. Furthermore, the craft-style panel were better at identifying the attributes in an identification test both before and after training, even though results were not significant. Additionally, only the craft-style panel had a significant increase between the number of correct identifications before and after training. This indicates that the craft-style panel had a higher attribute knowledge compared to the non-drinker panel and that the training sessions were more efficient for the craft-style panel. The results from the perceptual similarity showed that a difference in sample sensory configurations was observed before the training sessions, but that this difference was not found after training. This indicates that the training sessions increased the perceptual similarity between the two panels. To sum up, the beer consumption habit influenced all aspects of panel performance before training, with the craft-style panel performing better than the non-drinkers panel. However, the panels’ performance became more similar after a short period of training sessions. Furthermore, the study concludes that product involvement should be measured on a product specific level (i.e., drinking habit), as compared to a more general level (i.e., FIS score).

## Figures and Tables

**Table 1:** Sample overview with specification of modifications.

#	Sample Name	Sample Modification	Sensory Profile Alterations
1	Control	Carlsberg pilsner with no modification	Unaltered
2	Bitter Low	Carlsberg pilsner + Isohop® (0.012 µL/mL)	Slight increased bitter taste
3	Bitter High	Carlsberg pilsner + Isohop® (0.024 µL/mL)	Intense increased bitter taste
4	Malt Low	Carlsberg pilsner + isobutyraldehyde (1 capsule ^1^/1500 mL)	Slight increased malty flavor
5	Malt High	Carlsberg pilsner + isobutyraldehyde (1 capsule ^1^/1000 mL)	Intense increased malty flavor
6	Fruity Low	Carlsberg pilsner + iso-amyl acetate (1 capsule ^2^/1300 mL)	Slight increased fruity flavor
7	Fruity High	Carlsberg pilsner + iso-amyl acetate (1 capsule ^2^/800 mL)	Intense increased fruity flavor
8	Sulfur Low	Carlsberg pilsner + hydrogen sulfphide (1 capsul e^3^/1300 mL)	Slight increased sulfury flavor
9	Sulfur High	Carlsberg pilsner + hydrogen sulphide (1 capsule ^3^/800 mL)	Intense increased sulfury flavor
10	Hoppy Low	Carlsberg pilsner + hop oil extract (1 capsule ^4^/1500 mL)	Slight increased hoppy flavor
11	Hoppy High	Carlsberg pilsner + hop oil extract (1 capsule ^4^/1000 mL)	Intense increased hoppy flavor

^1^ 80 µg isobutyraldehyde per capsule, ^2^ 3.5 mg isoamyl acetate per capsule, ^3^ 18 µg hydrogen sulphide per capsule, ^4^ 1.25 mg hop oil extract per capsule.

**Table 2:** Chi-squared tests based on number of correct versus incorrect attribute identifications, with Yates correction. Significant differences are marked in bold.

Product	Craft Vs. Non			
	Pre		Post	
	χ^2^	*p* Value	χ^2^	*p* Value
Bitter Low	0.01	0.941	5.24	**0.022**
Bitter High	0.41	0.522	0.00	1.000
Malt Low	2.42	0.120	1.12	0.290
Malt High	0.00	1.000	0.01	0.941
Fruit Low	0.04	0.840	0.50	0.478
Fruit High	2.08	0.149	0.18	0.676
Sulfur Low	0.06	0.811	2.83	0.092
Sulfur High	0.42	0.518	0.67	0.413
Hoppy Low	0.44	0.507	3.59	**(0.058)**
Hoppy High	0.00	1.000	3.23	0.072

## Appendix A. Overview of the Screening Questionnaire

**Table A1:** Overview of the screening questionnaire content.

#	Question Formulation	Answer Options
1	*Please provide your first and last name:*	written answer
2	*Please provide your email address:*	written answer
3	*Please indicate your gender:*	Male/Female
4	*Please provide your age:*	Numerical value
5	*What is your current occupation?*	A. Employed full time B. Employed part time C. Unemployed looking for work D. Unemployed not looking for work E. Retired F. Student G. Disabled
6	*Approximately, How many sensory studies have you participated in?*	Numerical value
7	*Do you drink beer regularly?*	Yes/No
8	*Please state why you do not drink beer?*	Written answer
9	*Would you be willing to taste?*	Yes/No
10	*Please choose the beer(s) you usually drink. Choose as many as you like. If the beer you usually drink is not present, please choose a similar style of beer.*	Pictures and names of the following beers were presented: A. Budweiser Light B. Budweiser **C. Lagunitas IPA** D. Coors Light **E. Stone IPA** F. Miller Light G. Pabst Blue Ribbon **H. Pliny the Elder** **I. Sierra Nevada Pale Ale**
11	*How interesting do you find brewing and beer production?*	A. Extremely interesting B. Very interesting C. Moderately interesting D. Slightly interesting E. Not interesting at all
12	*Do you or any of your close relatives work with beer professionally?*	Yes/No

Italic text represents the questions asked. Question 8 and 9 were only displayed if the answer for question 7 was *No*. Question 10 was only displayed if the answer for question 7 was *Yes.* In question 10, the beers in bold are craft-style beers, while the non-bold beers are light-style. Furthermore, the presentation order of the beers was randomized.

## Appendix B. The Food Involvement Scale

**Table A2:** Overview of the different items included in the food involvement scale (FIS, [28]) and whether the score for each item should be reversed during calculation of the final FIS score. Participants are asked to indicate how much they agree with the statement in each item on a 7-point scale with labeled endpoints named disagree strongly to agree strongly.

#	Reversed	Food Involvement Scale Item
1	x	I don’t think much about food each day
2	x	Cooking or barbequing is not much fun
3		Talking about what I ate or am going to eat is something I like to do
4	x	Compared with other daily decisions, my food choices are not very important
5		When I travel, one of the things I anticipate most is eating the food there
6		I do most or all of the clean up after eating
7		I enjoy cooking for others and myself
8	x	When I eat out, I don’t think or talk much about how the food tastes
9	x	I do not like to mix or chop food
10		I do most or all of my own food shopping
11	x	I do not wash dishes or clean the table
12		I care whether or not a table is nicely set
